# Antihyperglycemic Effect of Ganoderma Lucidum Polysaccharides on Streptozotocin-Induced Diabetic Mice

**DOI:** 10.3390/ijms12096135

**Published:** 2011-09-20

**Authors:** Fenglin Li, Yiming Zhang, Zhijian Zhong

**Affiliations:** 1 Department of Bioengineering, Collage of Jilin Agricultural Science and Technology, Jilin 132101, Jilin, China; 2 College of Enviroment & Chemical Engineering, Yanshan University, Qinhuangdao 066400, Hebei, China; 3 Department of Plastic, Cosmetic and Reconstructive Surgery, Xinqiao Hospital, Chongqing 400037, China; E-Mail: zympla@sina.com; 4 Department of Arctic and Marine Biology, Faculty of Biosciences, Fisheries and Economics, University of Troms, Troms 9037, Norway; E-Mail: 568169115@qq.com

**Keywords:** antihyperglycemic, ganoderma lucidum polysaccharides, streptozotocin, diabetic mice

## Abstract

The current study evaluated the glucose-lowering effect of ganoderma lucidum polysaccharides (Gl-PS) in streptozotocin (STZ)-induced diabetic mice. The diabetic mice were randomly divided into four groups (8 mice per group): diabetic control group, low-dose Gl-PS treated group (50 mg/kg, Gl-PS), high-dose Gl-PS treated group (150 mg/kg, Gl-PS) and positive drug control treated group (glibenclamide, 4 mg/kg), with normal mice used as the control group. Body weights, fasting blood glucose (FBG), serum insulin and blood lipid levels of mice were measured. After 28 days of treatment with Gl-PS, body weights and serum insulin levels of the Gl-PS treated groups was significantly higher than that of the diabetic control group, whereas FBG levels was significantly lower. Moreover, total cholesterol (TC), triglyceride (TG) and low density lipoprotein cholesterol (LDL-C) levels of the Gl-PS treated groups had dropped, whereas the high density lipoprotein cholesterol (HDL-C) levels had increased. In addition, according to acute toxicity studies, Gl-PS did not cause behavioral changes and any death of mice. These data suggest that Gl-PS has an antihyperglycemic effect. Furthermore, considering the Gl-PS effects on lipid profile, it may be a potential hypolipidaemic agent, which will be a great advantage in treating diabetic conditions associated with atherosclerosis or hyperlipidemia.

## 1. Introduction

Diabetes mellitus (DM) is a metabolic disorder characterized by hyperglycemia and alterations in carbohydrate, fat, and protein metabolism, associated with absolute or relative deficiencies in insulin secretion and/or insulin action. The condition is associated with several complications such as atherosclerosis, neuropathy, and cataract formation [1–4]. Diabetes mellitus is considered a “modern day epidemic” and is rightly recognized as a global public health issue. The number of adults with diabetes in the world will rise to 300 million by the year 2025 and the major part of this numerical increase will occur in developing countries [5]. It can be classified into type I diabetes (insulin-dependent diabetes mellitus) and type II diabetes (non-insulin-dependent diabetes mellitus). Type II diabetes accounts for about 90% of the disease, and is characterized by peripheral insulin resistance and impaired insulin secretion, which is often associated with lipid and lipoprotein disorders [6–8]. Many oral hypoglycemic agents, such as biguanides, are available for the treatment of diabetes [9], but these synthetic agents are associated with drawbacks such as rigid and multiple dosing regimen, high-cost, inaccessibility and untoward effects [10]. These factors have contributed to the recent increase in the use of folkloric plant products.

Ganoderma lucidum is a favorite remedy in oriental medicine for centuries. Its fruiting body is called “Lingzhi” in China and “Reishi” in Japan. It has been known as a traditional remedy, used in Chinese and Japanese traditional medicine for treatment of several diseases, such as hepatitis, hypertension, chronic bronchitis, bronchial asthma, cancer and others [11,12]. Besides, in some places, such as Jilin province and Shangdong province of China, ganoderma lucidum water decoction has been used as folk medicine to treat diabetes mellitus. During the past 20 years, reports have shown that ganoderma lucidum extracts have beneficial therapeutic effects on diabetes. Seto *et al.* [13] report that ganoderma lucidum water extracts can provide beneficial effects in treating type II diabetes by lowering the serum glucose levels through the suppression of the hepatic PEPCK gene expression. Mohammed *et al.* [14] report that the Ethylacetate and n-Butanol fractions of ganoderma lucidum aqueous extracts have potent anti-diabetic effects. The Ethylacetate fraction has no significant erythropoetic effect while the n-Butanol fraction shows a significant decrease in the red cell parameters. Jung *et al.* [15] report that ganoderma lucidum may stimulate glucose uptake, through both PI 3-kinase and AMPK in L6 skeletal muscle cells thereby contributing to glucose homeostasis. The major bioactive components in ganoderma lucidum are polysaccharides (Gl-PS), ganoderic acid (triterpene), and adenosine, while the Gl-PS are the major source of its biological activity and therapeutic use [16–20]. Several studies have shown that Gl-PS possess anti-oxidant, anti-tumor, immunomodulatory and immunotherapeutic activities [16,21–24]. Although ganoderma lucidum extracts have already shown anti-diabetic effects in some researches, there are few studies on therapeutic effects on diabetes of Gl-PS. Therefore, the aim of this study was to investigate the glucose-lowering effect of Gl-PS in streptozotocin (STZ)-induced diabetic mice.

## 2. Results and Discussion

### 2.1. Acute Toxicology Test of Gl-PS in Mice

Acute toxicology test of Gl-PS was evaluated in mice at doses up to 5000 mg/kg body weight p.o. administered for 48 h. Gl-PS did not cause behavioral changes and no death was observed. The oral LD_50_ value of Gl-PS was greater than 5000 mg/kg bodyweight in mice and considered to be a practically non-toxic substance.

### 2.2. Effects of Gl-PS on Body Weights in Mice

**Figure 1:**
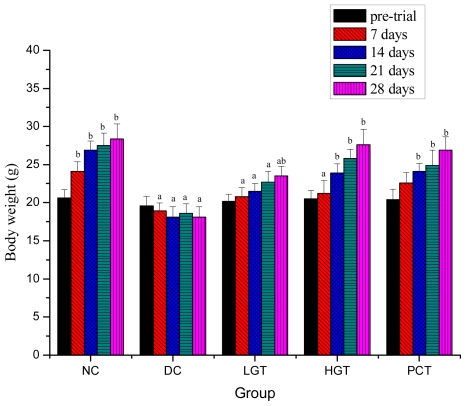
Effects of Gl-PS on body weights in mice. Data were presented as means ± SD. NC group: normal control group, DC group: diabetic control group, LGT group: low-dose Gl-PS (50 mg/kg) treated group, HGT group: high-dose Gl-PS (150 mg/kg) treated group, PCT group: positive drug (4 mg/kg, glibenclamide) control treated group. ^a^ *P <* 0.05 as compared with NC group. ^b^ *P <* 0.05 as compared with DC group.

Effects of Gl-PS on body weights in mice are shown in Figure 1. Before experiment, body weights were not significantly different between groups. After 7 days of treatment with Gl-PS, body weights of the low-dose Gl-PS treated group (LGT), high-dose Gl-PS treated group (HGT) and diabetic control group (DC) groups were significantly lower (*P <* 0.05) than that of the normal control group (NC) group, 13.8%, 15.9% and 27.5% lower, respectively. However, body weights of HGT group were significantly elevated by 32.1%, compared to that of DC group after 14 days (*P <* 0.05). After 28 days of treatment with Gl-PS, body weights of the LGT and HGT groups was significantly higher (*P <* 0.05) than that of the DC group, 29.8% and 58.1% higher, respectively. And there was no significant difference between the NC and HGT groups.

STZ is a valuable agent for experimental induction of diabetes. Its diabetogenic effect is the direct result of irreversible damage to the pancreatic beta cells, resulting in degranulation and loss of insulin secretion [23]. Insulin deficiency will lead to decreased activity of lipoprotein lipase and increased mobilisation of free fatty acids from peripheral fat depots. The STZ-induced diabetic animal is thus considered as an animal model of type I diabetes and hyperlipidemia [24]. STZ-induced diabetes was characterized by a severe loss in body weight, which has also been reported by other researchers [25,26], and this reduction in body weight is due to the loss or degradation of structural proteins, since structural proteins are known to contribute to the body weight [27]. Previous reports show that protein synthesis is decreased in all tissues due to decreased production of ATP and absolute or relative deficiency of insulin [27,28]. In the present study, when diabetic mice were treated with Gl-PS, the weight loss improved, which might be as a result of its ability to reduce hyperglycemia

### 2.3. Effects of Gl-PS on Fasting Blood Glucose (FBG) and Serum Insulin Levels in Mice

**Figure 2:**
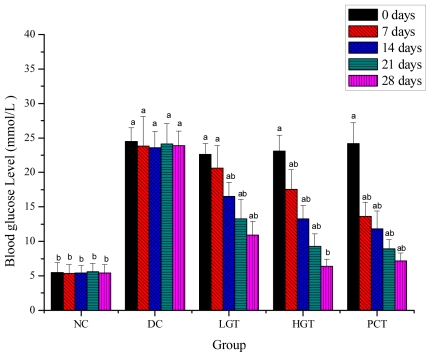
Effects of Gl-PS on fasting blood glucose levels in mice. Data were presented as means ± SD. NC group: normal control group, DC group: diabetic control group, LGT group: low-dose Gl-PS (50 mg/kg) treated group, HGT group: high-dose Gl-PS (150 mg/kg) treated group, PCT group: positive drug (4 mg/kg, glibenclamide) control treated group. ^a^ *P <* 0.05 as compared with NC group. ^b^ *P <* 0.05 as compared with DC group.

**Figure 3:**
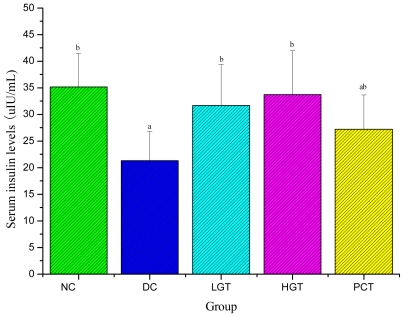
Effects of Gl-PS on serum insulin levels in mice. Data were presented as means ± SD. NC group: normal control group, DC group: diabetic control group, LGT group: low-dose Gl-PS (50 mg/kg) treated group, HGT group: high-dose Gl-PS (150 mg/kg) treated group, PCT group: positive drug (4 mg/kg, glibenclamide) control treated group. ^a^ *P <* 0.05 as compared with NC group. ^b^ *P <* 0.05 as compared with DC group.

Effects of Gl-PS on FBG and serum insulin levels in mice are shown in Figures 2 and 3. As shown in Figure 2, FBG levels in NC group maintained constant during the experimental period and was significantly lower than diabetic groups before experiment (*P <* 0.05). In the LGT, HGT and PCT groups, FBG levels showed a decreasing trend. When compared to the DC group, significant differences were seen for the LGT and HGT groups in the 14th day (*P <* 0.05), 43.0% and 78.1% lower, respectively. After 28 days of treatment with Gl-PS, there was no significant difference between the NC and HGT groups. As shown in Figure 3, serum insulin levels of DC groups were significantly lower than that of the NC group. After 28 days of treatment with Gl-PS, serum insulin levels of the LGT and HGT groups were significantly higher (*P <* 0.05) than that of the DC group, 48.2% and 27.8% higher, respectively.

Effective control of the blood glucose level is a key step in preventing or reversing diabetic complications and improving the quality of life diabetic patients [29–31]. The present study shows that Gl-PS produced a significant drop in FBG levels in diabetic mice and the dosage of 150 mg/kg is more effective than that of 50 mg/kg. One of the possible mechanisms of this antihyperglycemic effect could be due to an insulin-release stimulatory effect. Therefore, Gl-PS on serum insulin levels on STZ-induced diabetic mice was investigated. In the present study, the data indicated that given Gl-PS the treatments induced an increment in serum insulin levels, which might increase the renewal of beta cells in the pancreas or permit the recovery of partially destroyed beta cells and stimulates pancreatic insulin secretion.

### 2.4. Effects of Gl-PS on Blood Lipid Levels in Mice

**Figure 4:**
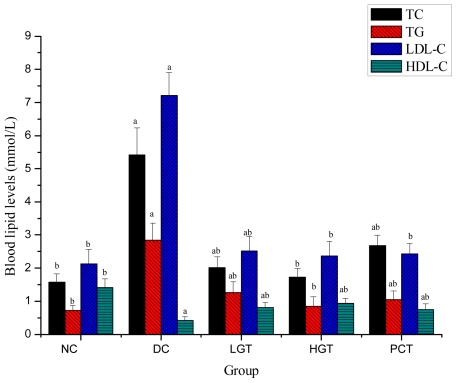
Effects of Gl-PS on blood lipid levels in mice. Data were presented as means ± SD. NC group: normal control group, DC group: diabetic control group, LGT group: low-dose Gl-PS (50 mg/kg) treated group, HGT group: high-dose Gl-PS (150 mg/kg) treated group, PCT group: positive drug (4 mg/kg, glibenclamide) control treated group. ^a^ *P <* 0.05 as compared with NC group. ^b^ *P <* 0.05 as compared with DC group.

Effects of Gl-PS on blood lipid levels in mice are shown in Figure 4. TC, TG and LDL-C levels of DC groups were significantly higher than that of the NC group, 244.6%, 289.1% and 238.5% higher, respectively. After 28 days of treatment with Gl-PS, TC levels of the LGT and HGT groups were significantly lower (*P <* 0.05) than that of the DC group, 150.7% and 212.7% lower, respectively. TG levels of the LGT and HGT groups were significantly lower (*P <* 0.05) than that of the DC group, 125.4% and 234.1% lower, respectively. LDL-C levels of the LGT and HGT groups were significantly lower (*P <* 0.05) than that of the DC group, 187.3% and 204.2% lower, respectively. And the levels of these parameters were resettled towards the control level. HDL-C levels of DC groups were significantly lower than that of the NC group, 235.7% higher. After 28 days of treatment with Gl-PS, HDL-C levels of the LGT and HGT groups were significantly higher (*P <* 0.05) than that of the DC group, 92.9% and 121.4% lower, respectively.

It is well known that dyslipidemia is associated with uncontrolled diabetes mellitus. The blood levels of TC, TG and LDL-C increase, while the HDL-C levels decline, contributing to secondary complications of diabetes [32–34]. In the present study, diabetic mice exhibited a significant elevation of TC, TG, and LDL-C, while HDL-C was decreased. After the Gl-PS supplementation resulted in lowering the TC, TG and LDL-C levels with elevation of HDL-C levels. The data indicated that Gl-PS may decrease the risk of cardiovascular disease and hasten removal of cholesterol from peripheral tissues to liver for catabolism and excretion. These effects may be due to low activity of cholesterol biosynthesis enzymes or low level of lipolysis which are under the control of insulin [35].

## 3. Experimental Section

### 3.1. Chemicals

A glucose analyzer (GT-1640) and glucose check strips were purchased from Arkray Inc. (Japan). STZ and glibenclamide were purchased from Sigma Chemical Co. (USA). Reagents for TG, HDL-C, LDL-C were purchased from Beijing Chengxinde Biochemistry Reagent Co. (Beijing, China). Reagents for TC were purchased from Nanjing Jiancheng Biochemistry Reagent Co. (Nanjing, China). Reagents for serum insulin were purchased from Beijing Beifang Pharmaceutical Co. (Beijing, China). All other chemicals were of the highest commercial grade available on the domestic market. The freshly prepared redistilled water was used in the present study.

### 3.2. Extraction of Gl-PS

The fruiting bodies of ganoderma lucidum were purchased from a local medicine shop in Jilin city, China. Sporocarps were cut into small pieces, dried at 40–50 °C for 48 h and powdered. Gl-PS were extracted by method of Wang *et al.* with slight modification [36]. Briefly, Gl-PS was extracted by hot water from the ganoderma lucidum fruiting body, followed by ethanol precipitation, dialysis, and protein depletion using the Sevag method. The total yield of Gl-PS was 0.82% (w/w) in terms of the ganoderma lucidum fruiting body. As a hazel-colored water-soluble powder, GL-PS was dissolved in distilled water and stored at 4 °C before use.

### 3.3. Animals

All experiments were performed in accordance with the Guide for the Care and Use of Laboratory Animals of the Chinese National Institutes of Health. Male Kunming mice weighing approximately 18 to 22 g were obtained from Jiuzhan Biochemical Factory (Jilin, China). The animals were housed in a room maintained at 23 ± 2 °C with relative air humidity of 45% to 55% on a 13-hour light/11-hour dark cycle. Mice were provided a standard laboratory chow and water *ad libitum*. The approval of this experiment was obtained from the Institutional Animal Ethics Committee of Jilin Agricultural Science and Technology (Jilin, China).

### 3.4. Acute Toxicology Test

Acute toxicology test in mice was performed according to the method of Chao *et al.* [37]. Male Kunming mice were divided into test and control groups (8 mice per group). The test was performed by using increasing oral doses of Gl-PS (500, 2500, and 5000 mg/kg body weight), in 10 mL/kg volume to different test groups. Control group received saline solution (10 mL/kg). The experimental mice were allowed for food *ad libitum*, were all kept under regular observation for 48 h, for any mortality or behavioral changes (irritation, restlessness, respiratory distress, abnormal locomotion and catalepsy).

### 3.5. Induction of Diabetes Mellitus

The mice were adapted to diet and environment for 1 week before the experiment began. After a 24-hour fasting, diabetes was subsequently induced in the mice through intraperitoneal (ip) administration of STZ at a dose of 100 mg/kg body weight. STZ was freshly prepared in an ice-cold citrate buffer (0.1 mmol/L, pH 4.5) and immediately injected into the animals (within 5 min). A week later, high and steady blood glucose levels were observed in STZ-induced mice. At this point, the STZ-induced mice with high blood glucose levels (*>*11.1 mmol/L) were selected as diabetic models [38]. Measurement of blood glucose levels was carried out by use glucose check strips.

### 3.6. Experimental Design

STZ-induced diabetic mice (mentioned above) were randomly divided into four groups (8 mice per group), and normal mice were used as the control group.

Group I (*n* = 8): normal control group (NC), normal mice were allowed to free access to a normal diet and treated with saline solution for 28 days.

Group II (*n* = 8): diabetic control group (DC), the diabetic mice were allowed to free access to a normal diet and treated with saline solution for 28 days.

Group III (*n* = 8): low-dose Gl-PS treated group (LGT), the diabetic mice were put on a normal diet and treated with 50 mg/kg of Gl-PS for 28 days.

Group IV (*n* = 8): high-dose Gl-PS treated group (HGT), the diabetic mice were put on a normal diet and treated with 150 mg/kg of Gl-PS for 28 days.

Group V (*n* = 8): positive drug control treated group (PCT), the diabetic mice were put on a normal diet and treated with 4 mg/kg of glibenclamide for 28 days.

In a preliminary experiment, the dose of Gl-PS used in this study was tried and confirmed to be suitable and effective in test mice. FBG levels were measured for once every week. Blood was collected from tip of the tail vein (starting from 9:00 a.m.) after a 12- to 14- hour overnight fast. At the same time, body weights of mice were measured using balance. On the last day of experiment, the mice were deprived of food overnight and sacrificed by cervical dislocation. Blood was collected in polystyrene tubes without the anticoagulant. Serum was immediately separated by centrifugation at 3000 rpm at room temperature for 10 min. Samples were stored at −20 °C for the assay of TC, TG, HDL-C, LDL-C and serum insulin. TC, TG, LDL-C and HDL-C were determined by enzyme methods, serum insulin level was estimated by insulin-ELISA kit according to the manufacturer’s instruction.

### 3.7. Statistical Analysis

All results are expressed as mean ± SD. for eight mice in each group. To determine the effect of treatment, data were analyzed using one-way ANOVA repeated measures. P-values of less than 0.05 were regarded as significant. Significant values were assessed with Duncan’s multiple range test. Data were analyzed using the statistical package “SPSS 12.0 for Windows”.

## 4. Conclusions

This study indicates that ganoderma lucidum polysaccharides (Gl-PS) have an antihyperglycemic effect. The possible mechanism of this antihyperglycemic effect is that Gl-PS might increase the renewal of beta cells in the pancreas or permit the recovery of partially destroyed beta cells and stimulates pancreatic insulin secretion. There are some studies that have shown that Gl-PS can reduce and delay the absorption of glucose in rats [39,40]. We need further studies to clarify the mechanisms. Considering the Gl-PS effect on lipid profile, it may be a potential hypolipidaemic agent, which will be a great advantage in treating diabetic conditions associated with atherosclerosis or hyperlipidemia.
